# Sarcolipin but not phospholamban responds to *in-vivo* heat stress in rat skeletal muscle similar to the 70-kDa heat shock protein

**DOI:** 10.3389/fphys.2026.1763668

**Published:** 2026-03-03

**Authors:** A. N. Brahmbhatt, J. Z. Chan, M. V. Tomczewski, A. Humaid, H. I. Gallichan, J. Flanagan, R. E. Duncan, A. R. Tupling

**Affiliations:** Department of Kinesiology & Health Sciences, University of Waterloo, Waterloo, ON, Canada

**Keywords:** 70-kDa heat shock protein, heat stress, phospholamban, Sarco(endo)plasmicreticulum Ca2+-ATPase, sarcolipin

## Abstract

Sarco/endoplasmic reticulum Ca^2+^ ATPase (SERCA) pumps are found on the membrane of the sarcoplasmic reticulum (SR) which actively transport Ca^2+^ from the cytosol into the SR. The 70-kDa heat shock protein (Hsp70), sarcolipin (SLN) and phospholamban (PLN) can preserve SERCA function in the face of heat stress (HS). However, it remains unknown if SLN and PLN can be stress-induced proteins like Hsp70. Therefore, the purpose of this study was to compare the SLN and PLN gene and protein time course (0, 24, and 48 h) response to *in-vivo* HS with that of Hsp70 in rat soleus (SOL) and white gastrocnemius (WG). The HS protocol involved submerging the lower limbs of the animals either in a 37 °C (control) or 44 °C–45 °C (HS) water bath for 30 min. We detected increases in *Hsp70* gene expression in the male WG immediately post-HS and in the female SOL after 48 h. Similarly, protein expression was induced after 24 h in both the muscles of males and in the female WG, and remained elevated after 48 h in the male SOL. SLN gene expression was induced in the male WG after 48 h and a trending increase was found for protein expression in the male SOL following HS. In contrast, PLN did not show any signs of stress-induction in either muscle or sex. These results suggest that like Hsp70, SLN, but not PLN, is a stress-induced protein that responds to *in-vivo* HS in a sex- and muscle-specific manner.

## 1 Introduction

In muscle, the sarcoplasmic reticulum bound sarco/endoplasmic reticulum Ca^2+^-ATPase (SERCA) pump is a major regulator of cytosolic Ca^2+^ levels (Gamu et al., 2020). SERCA is a 110 kDa P-type ATPase that induces muscle relaxation by transporting Ca^2+^ against its concentration gradient in an ATP-dependent manner into the SR lumen (Gamu et al., 2020; Gamu et al., 2014; Rossi and Dirksen, 2006). In diseased states, SERCA is often damaged, and its function is consequently impaired which then further contributes to calcium dysregulation (Valentim et al., 2022; Gailly et al., 2007; Murphy et al., 2006; Goonasekera et al., 2011). Therefore, the preservation of SERCA function in the face of physiological stress is essential in allowing for proper regulation of cytosolic Ca^2+^ levels and thus preserving the health and function of muscle.

Structurally, SERCA is composed of 3 regions: a cytosolic region which contains the phosphorylation (P-domain), actuator (A-domain) and nucleotide-binding (N-domain); a transmembrane (TM) region, and a luminal region (Toyoshima et al., 2000). Within the TM region lies the two Ca^2+^ binding sites (Lee and East, 2001). The two most widely studied inhibitors of SERCA are sarcolipin (SLN) and phospholamban (PLN). SLN and PLN are 31 and 52 amino acids in length, respectively, and display significant sequence homology within their transmembrane regions (Bhupathy et al., 2007). Accordingly, both proteins inhibit SERCA by interacting with its Ca^2+^ binding sites.

Several studies have reported the impact of stress on SERCA structure and function (Tupling et al., 2004; Fu and Tupling, 2009; Fu et al., 2020; Gutierrez-Martin et al., 2004; Klebl et al., 1998; Senisterra et al., 1997; Viner et al., 1999). SERCA is susceptible to oxidative/nitrostative damage in the face of heat shock which impairs its function (Fu and Tupling, 2009; Fu et al., 2020). The 70-kDa heat shock protein (Hsp70) is a stress-induced chaperone protein that is highly sensitive to changes in redox state, protein oxidation, and increased levels of unfolded proteins caused by cellular stress (Kalmar and Greensmith, 2009; Larkins et al., 2012; Liu and Steinacker, 2001). We have shown in both SR membrane fractions (Tupling et al., 2004) and in HEK293 cells (Fu and Tupling, 2009), that Hsp70 preserves SERCA’s N-domain structure and function in the face of cellular stress.

However, this protection subsides with increased severity of stress, and it was postulated that this may be because the transmembrane region, which is inaccessible to Hsp70, was being denatured (Tupling et al., 2004). SLN and PLN are two proteins that interact with SERCA within the transmembrane region and so it was hypothesized that they may provide protection to the pump in this region (Fu et al., 2020). *In vitro* experiments in HEK293 cells showed that SERCA maximal activity was preserved in the face of a 60 min exposure to heat stress (HS) if either SLN or PLN were present (Fu et al., 2020). *Ex-vivo* experiments using left ventricle and diaphragm also demonstrated that endogenous expression of SLN and PLN preserves maximal SERCA activity in the face of both a 30 and 60 min exposure to HS (Fu et al., 2020). The functional protection these proteins provide is much like that of Hsp70, but it has yet to be observed whether SLN and PLN are stress-inducible proteins like Hsp70. Therefore, the primary purpose of this study was to assess the SLN and PLN gene and protein time course (0, 24, and 48 h) response to *in-vivo* HS in rat skeletal muscle and compare it with that of Hsp70. We also investigated this response in male and female rats to determine whether it differs by biological sex.

## 2 Methods

### 2.1 Animals

All experiments were performed on 8–12 week-old Sprague Dawley rats who were randomly assigned to 4 conditions - control (CTL), and 3 HS conditions, in which animals were either allowed no recovery and were euthanized immediately after treatment (HS0), or were allowed to recover for 24 h (HS24), or 48 h (HS48) post-treatment (n = 12/condition). Each condition had a male and female group to allow for sex specific assessments (n = 6/sex within each condition). From these groups, soleus (>95% type I fibres) and white gastrocnemius (100% type II fibres) (Bloemberg and Quadrilatero, 2012) muscles were excised and processed for experimental analyses. All experiments within this study were reviewed and approved by the University of Waterloo Animal Care Committee in accordance with the Canadian Council on Animal Care.

### 2.2 *In-Vivo* heat stress

In all conditions, animals were first anesthetized in an induction chamber with 4%–5% isoflurane and were then transferred to the water bath. Only the lower limbs were submerged in the water bath and the isoflurane dose was reduced to 1.5%–2.5% throughout the treatment period. The control animals underwent treatment at 37 °C for 30 min with their core temperature (assessed with a rectal probe) being maintained at 37 °C ± 0.5 °C, and the tissues of interest were harvested immediately afterwards while the animal was still under anaesthesia and were subsequently euthanized by CO_2_ exposure (20%–30% flow rate of chamber volume per minute). The *in-vivo* HS protocol employed in this study has been previously used to induce an Hsp70 response (King et al., 2002; Tolson and Roberts, 2005). The 3 HS conditions involved submerging the lower limbs of the anesthetized rats in a water bath set at a temperature between 44 °C and 45 °C and core temperature was maintained between 41 °C and 41.5 °C (HS) for 30 min. After either no recovery or a recovery period of either 24 or 48 h, the animals were once again anesthetized for tissue harvesting and were subsequently euthanized by CO_2_ exposure. To ensure the animals’ safety, a pulse oximeter, which was attached at their front paw, was used to monitor their blood oxygen levels and heart rate. They were also provided with a 2–3 mL subcutaneous injection of saline for rehydration after the HS protocol.

### 2.3 Total protein oxidation

Total protein oxidation was measured using a previously described technique (Hughes et al., 2024; Bellissimo et al., 2023) which uses a maleimide tagged infrared fluorescent dye that binds to free sulfhydryl (SH) groups. Samples were first processed via Zeba Desalting Spin Columns (Thermo Fisher Scientific, Burlington, Canada) to remove glutathione and free cysteines and protein concentration was quantified using a BCA (Thermo Fisher Scientific, Burlington, Canada) assay. The samples were then diluted to 1 μg/μL and incubated with IRDye 800CW-Maleimide (LiCor Biotechnology, Lincoln, NE, United States) overnight at 4 °C at a concentration of 100 nM/200 µg of protein. Samples were processed once again via Zeba Desalting Spin Columns to remove excess dye, and protein concentration was reassessed. 2.5 µg of each sample was then separated electrophoretically using standard SDS-PAGE (12% total acrylamide) at 160 V for 75 min and subsequently imaged at 800 nm emission wavelengths using a ChemiDoc Imaging System (Bio-Rad, Hercules, CA, United States).

### 2.4 Western blotting

Muscles samples were homogenized 1:10 (w/v) in ice-cold homogenizing buffer (250 mM sucrose, 5 mM HEPES, 0.2 mM PMSF and 0.2% [w/v] NaN3) using a 5 mL glass homogenizer and stored at −80 °C. Subsequently, these muscle homogenates were solubilized into a 1x buffer, and the proteins were electrophoretically separated with the use of a tricine based SDS-PAGE (13% total acrylamide for PLN and SLN) and a standard SDS-PAGE (7.5% total acrylamide for Hsp70). The separated proteins were transferred either via a semi-dry (Hsp70) or wet-transfer (SLN and PLN) onto a polyvinylidene difluoride membrane (PLN and Hsp70) or nitrocellulose membrane (SLN). Hsp70 (mouse, Stressgen Bioreagents, SPA-810, 1:1000) SLN (rabbit, Millipore Sigma, ABT13, 1:100), and PLN (mouse, ThermoFisher Scientific, MA3-922, 1:1000) proteins were immunoprobed with the corresponding primary antibodies and then immunoprobed with horseradish peroxidase-conjugated secondary antibodies. Luminata ForteTM was utilized to detect the resultant antigen-antibody complexes for Hsp70, whereas ECL Western blot Substrate (BioVision) was used to visualize SLN and PLN. ChemiDoc Imaging System (Bio-Rad, Hercules, CA, United States) was utilized to quantify the resulting optical densities which were normalized using ponceau stained proteins in the 37–50 kDa region. This region was selected because in our experience, this is a stable and protein dense region that consistently transfers and is highly reproducible across membranes.

### 2.5 RNA extraction, reverse transcription (RT) and real-time (q) PCR

Rat gastrocnemius and soleus muscles were homogenized separately in TRIzol® Reagent (Invitrogen, Waltham, MA) (1 mL/≤100 mg tissue), by mechanical disruption using a Polytron homogenizer, followed by sonication, and total RNA was obtained according to the manufacturer’s instructions. RNA samples were quantified using a Nanodrop 2000 Spectrophotometer and 2 mg of RNA was used to synthesize cDNA using a High-Capacity complementary DNA (cDNA) Reverse Transcription Kit according to the manufacturer’s protocol (Invitrogen, Waltham, MA), and was diluted 1:4 before storage at −80 °C until use.

For the real-time quantitative PCR assay, 2 µl of diluted cDNA was combined with 5 µl of AccuStart PCR ToughMix® mastermix (Quanta Bio, Beverly, MA), 0.5 µl of TaqMan® gene expression assay (Thermo Fisher Scientific) specific to the gene of interest (see Supplementary Table S1), and 2.5 µl of ddH_2_O, for a final reaction volume of 10 µl. The cycling parameters were the same for all genes: an initial cycle at 50 °C for 2 min, followed by another cycle at 90 °C for 20 s, followed by 49 cycles at 95 °C for 3 s, and finally, 60 °C for 30 s. Ct values of target genes were first normalized to β-Actin generating ΔCt measurements, and the relative gene expression levels in the experimental groups were determined using the ΔΔCt method, with the control group serving as the reference (Brahmbhatt et al., 2025).

### 2.6 Statistics

The data are presented as means ± SEM. GraphPad Prism was used for statistical analysis and graphical representation of data. Following ROUT testing for outliers, all data passed the Shapiro-Wilk test for normality. For each muscle, we ran either an unpaired t-test or a one-way ANOVA with sexes separated and combined. Where significant differences were found, Tukey’s post hoc test was used to compare specific means. Statistical significance was set at p < 0.05.

## 3 Results

### 3.1 Protein oxidation

**FIGURE 1:**
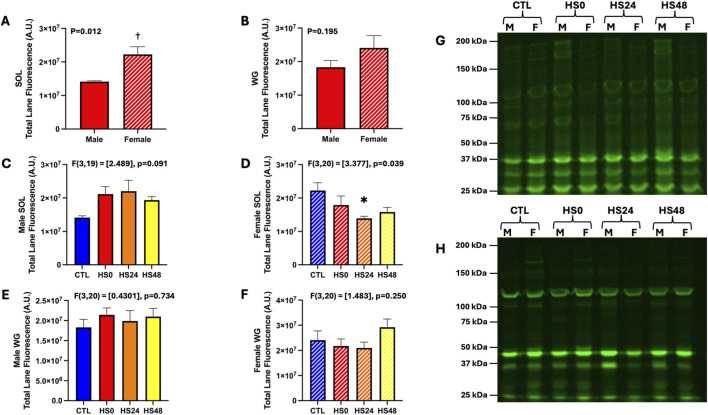
Total protein oxidation under basal conditions and following HS as assessed by IRDye 800CW bound maleimide in male and female rat soleus (SOL) and white gastrocnemius (WG). **(A,B)** Comparison of basal total protein oxidation between male and female SOL **(A)** and WG **(B)**; n = 5-6 per group. **(C–F)** Total protein oxidation in control and HS groups across 3 timepoints in male and female SOL [**(C,D)**, respectively] and WG [**(E,F)**, respectively]; n = 5-6 per group. **(G,H)** Representative blots from male and female rat SOL **(G)** and WG **(H)**. Protein oxidation status is reported as total lane fluorescence of the IRDye 800CW bound maleimide to free (reduced) sulfhydryl groups. Unpaired t-test **(A,B)** and one-way ANOVA **(C–F)** was performed and all data are presented as mean ± SEM. (†) p < 0.05 vs. male. (*) p < 0.05 vs. CTL.

To test the effectiveness of the HS protocol in generating oxidative stress within skeletal muscle, we assessed total protein oxidation and Hsp70 gene and protein expression as indicators of oxidative stress. Total protein oxidation was measured using a maleimide tagged infrared fluorescent dye which binds to free sulfhydryl groups. Theoretically, the oxidative stress generated by HS would reduce the number of free sulfhydryl groups and as a result there would be less maleimide binding compared with a more reduced environment expected in CTL. When comparing the basal protein oxidation status between males and females, it appears to be lower in the soleus from females compared with males (Figure 1A; p = 0.012), whereas in the white gastrocnemius (WG), although a similar pattern was seen, the differences were not statistically significant (Figure 1B; p = 0.195). While sex specific effects are part of our primary focus, we also performed a sex combined analysis to best capture a whole population effect. No effect of HS was observed at any timepoint in either muscle (Supplementary Figure S1; soleus–p = 0.456; WG–p = 0.339). However, when analysed with the sexes separated, we found a sex and muscle specific difference. As shown in Figure 1, HS did not change the total protein oxidation status at any timepoint in the male soleus (Figure 1C; p = 0.091), but it significantly increased total protein oxidation in the female soleus after 24 h compared with CTL (Figure 1D; p = 0.039). Total protein oxidation in WG was not different between CTL and HS across any timepoints in either males (Figure 1E; p = 0.734) or females (Figure 1F; p = 0.250).

### 3.2 Sex and muscle differences in Hsp70 expression

**FIGURE 2:**
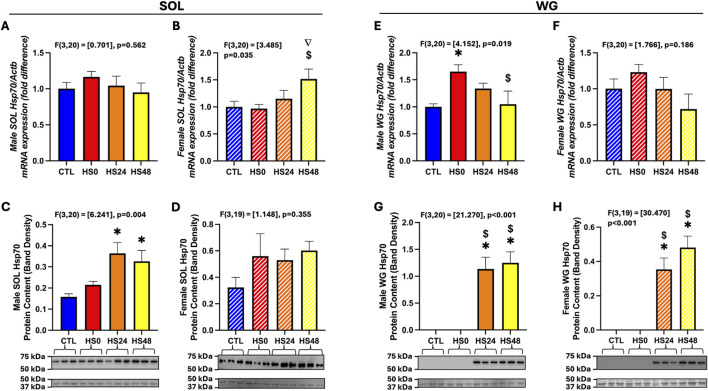
Hsp70 gene and protein response to HS in male and female rat soleus (SOL) and white gastrocnemius (WG). **(A–D)** Male and female SOL Hsp70 gene (n = 6 per group) [**(A,B)**, respectively] and protein [**(C,D)**, respectively] response (n = 5-6 per group). **(E–H)** Male and female WG Hsp70 gene (n = 6 per group) [**(E,F)**, respectively] and protein [**(G,H)**, respectively] response (n = 5-6 per group). One-way ANOVA was performed and all data are presented as mean ± SEM. Gene expression data are represented as fold difference relative to control (CTL). Representative western blots are shown with ponceau staining for proteins in the 37–50 kDa range used for normalization. (∇) p = 0.06 vs. CTL. ($) p < 0.05 vs. HS0. (*) p < 0.05 vs. CTL.

*Hsp70* (HSPA1A) gene expression was not different across timepoints in the soleus of both males and females combined (Supplementary Figure S2A; p = 0.827). Likewise, it was also not different in the male soleus (Figure 2A; p = 0.562), but in the female soleus, *Hsp70* gene expression (p = 0.035) was trending higher after 48 h relative to CTL (p = 0.060) and was significantly higher relative to HS0 (Figure 2B, p = 0.042). As for protein expression, it was significantly elevated in the soleus of both males and females combined after 24 (p = 0.012) and 48 (p = 0.008) hours relative to CTL (Supplementary Figure S2D). This increase was driven by changes observed in the male soleus at the same timepoints relative to CTL (Figure 2C; 24 h–p = 0.006; 48 h–p = 0.027). In contrast, Hsp70 protein expression was not significantly different across the timepoints in the female soleus (Figure 2D; p = 0.312). In the WG of males and females, Hsp70 gene expression was unchanged in response to HS across all timepoints (Supplementary Figure S2F; p = 0.095). In the male WG, *hsp70* gene expression was elevated at HS0 (p = 0.025) and returned to basal levels after 48 h (Figure 2E; p = 0.042). However, *hsp70* gene expression was not induced following HS in the female WG (Figure 2F; p = 0.186). Hsp70 protein expression was undetectable in male and female WG combined in the CTL and HS0 groups but was induced by HS and detectable after 24 (p < 0.001) and 48 h (Supplementary Figure S2H; p < 0.001). Analysed with the sexes separated, this response was also observed in the WG of both sexes (Figures 2G,H; males – p < 0.001 at both time points, females – p < 0.001 at both timepoints).

### 3.3 Sex and muscle differences in sarcolipin and phospholamban expression

**FIGURE 3:**
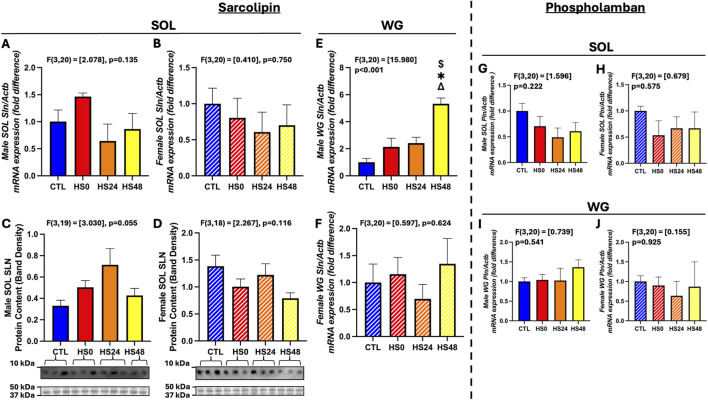
SLN and PLN gene and protein response to HS in male and female rat soleus (SOL) and white gastrocnemius (WG). **(A–D)** Male and female SOL SLN gene (n = 6 per group) [**(A,B)**, respectively] and protein [**(C,D)**, respectively**]** response (n = 5-6 per group). **(E,F)** Male **(E)** and female **(F)** WG SLN gene response (n = 6 per group). **(G–J)** Male and female SOL [**(G,H)**, respectively] and WG [**(I,J)**, respectively] PLN gene response (n = 6 per group). One-way ANOVA was performed and all data are presented as mean ± SEM. Gene expression data are represented as fold difference relative to control (CTL). Representative western blots are shown with ponceau staining for proteins in the 37–50 kDa range used for normalization. ($) p < 0.05 vs. HS0. (*) p < 0.05 vs. CTL. (Δ) p < 0.05 vs. HS24.

The primary purpose of this study was to examine the SLN and PLN gene and protein expression responses to HS. Sex-combined (Supplementary Figure S2B, p = 0.677) and sex-separated (Figures 3A,B; male – p = 0.135; female – p = 0.750) analysis did not reveal any *Sln* gene expression changes in response to HS in any of the groups. Likewise, sex-combined analysis of SLN protein expression was not significantly different across any of the timepoints (Supplementary Figure S2E; p = 0.254). However, when separated by sex, SLN protein response was different. In male soleus, it was trending higher following HS (Figure 3C; p = 0.055). Conversely, it was unchanged in the female soleus (Figure 3D, p = 0.116). In the WG of both males and females combined, *Sln* gene expression was significantly elevated at 48 h relative to CTL (Supplementary Figure S2G; p = 0.043). This change was driven by gene response observed in the male WG (Figure 3E; p < 0.001) which showed a significant increase after 48 h relative to all other timepoints but not female (Figure 3F; p = 0.624). Despite detecting *Sln* gene expression in the WG of both sexes, we were not able to detect SLN protein expression in any WG sample. As for *Pln* gene expression (Supplementary Figure S2I; Figures 3G–J), there were no differences between any groups in either muscle (p > 0.050) or sex (p > 0.050). We were also unable to detect PLN protein expression in either the soleus or WG regardless of sex or condition.

## 4 Discussion

**FIGURE 4:**
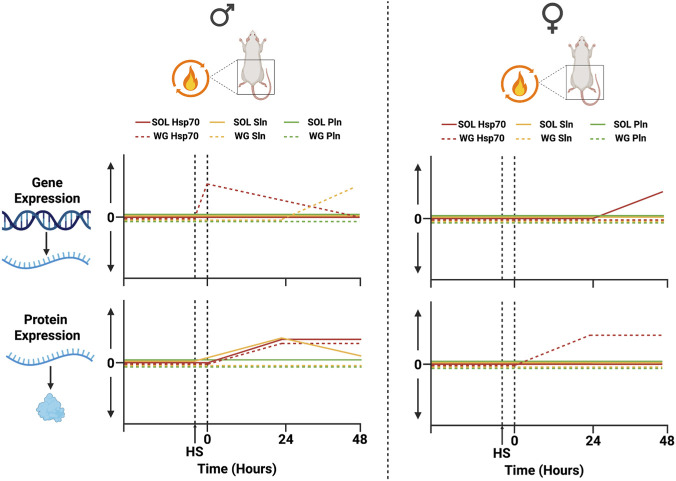
Summary of Hsp70, SLN, and PLN gene and protein time course response to HS in male and female rat soleus (SOL) and white gastrocnemius (WG). Gene and protein responses are displayed as changes from baseline immediately following HS (0 h) and 24 to 48 h post. Changes are not to scale. Made using Biorender.

The purpose of this study was to assess the time-course pattern of gene and protein expression of Hsp70, SLN, and PLN in the male and female rat soleus and white gastrocnemius in response to a physiological stressor and the main findings are summarized in Figure 4. The *in-vivo* heat stress protocol employed in this study i) induced gene and protein expression of Hsp70 and SLN in a sex- and muscle-specific manner, and ii) did not induce *Pln* gene or protein expression in either muscle of either sex.

Heat has been shown to produce oxidative stress (Bruskov et al., 2002; Flanagan et al., 1998) and is a widely used model used to study cellular stress (King et al., 2002; Tolson and Roberts, 2005; Demirel et al., 2003; Gupte et al., 1985; Von Schulze et al., 2021). Therefore, we expected that the intracellular environment in soleus and WG muscles would be oxidized immediately following HS. Surprisingly, total protein oxidation was only increased 24 h after HS in the soleus from female rats. Hsp70 is another hallmark stress response marker (Kalmar and Greensmith, 2009), which we found to be upregulated in both soleus and WG and in both males and females 24 and 48 h following HS. Collectively, these measures indicate that our experimental protocol induced oxidative stress that was relatively mild and less than originally intended, thereby limiting our assessment of HS effects on SLN and PLN gene and protein expression in muscle to the context of mild stress. It remains possible that a more severe stress model would have elicited different responses.

A consistent pattern across all measurements is that the time-course response to the HS employed in this study was muscle and sex specific. In the soleus, *Hsp70* gene expression was only found to be upregulated by HS in females, whereas Hsp70 protein expression appears to be elevated only in the males. In comparison, while *Sln* gene expression in soleus did not change following HS at any timepoint in both sexes, SLN protein expression appears to be increased in males but not females following HS. In the WG, *Hsp70* gene expression was only elevated in the males immediately following HS, whereas Hsp70 protein expression was elevated in both males and females after 24 h and remained elevated after 48 h. Despite loading 100 µg of protein, we could not detect SLN protein expression in the WG, suggesting that SLN may not be normally expressed in this muscle, as reported previously (Babu et al., 2007). Regardless, *Sln* gene expression in WG was upregulated 48 h following HS in males but not females. Within this context of only mild heat stress, our findings suggest that the responses of Hsp70 and SLN reflect mechanisms that may differ between muscle types and sexes.

The heat shock response is well characterized. Under basal conditions, Hsp70 remains bound to heat shock factor 1 (HSF-1) in the cytosol, but upon exposure to stress it dissociates to perform its chaperone role, binding and repairing damaged/unfolded proteins (Kalmar and Greensmith, 2009; Liu and Steinacker, 2001; Noble et al., 2008). The release of HSF-1 allows it to trimerize and translocate into the nucleus where it can bind heat shock elements (HSE) in nuclear DNA, thereby upregulating Hsp70 gene and subsequent protein expression (Kalmar and Greensmith, 2009; Liu and Steinacker, 2001; Noble et al., 2008). Our findings in the male WG, are consistent with this model, as both gene and protein expression of Hsp70 were elevated following HS. After sufficient Hsp70 protein is produced to handle the stressor, mRNA translation is expected to reduce, leading to mRNA transcript degradation (Silver et al., 2012; Masser et al., 2019; Krakowiak et al., 2018; Kmiecik et al., 2020). This would explain the reduced *Hsp70* gene expression after 48 h in the male WG. Perhaps, the same is not observed in the female soleus due to a delayed rise in gene and protein expression that may remain elevated beyond the 48 h timepoint. Notably, in the male soleus and female WG, Hsp70 protein expression increased despite no corresponding rise in gene expression, suggesting that post-transcriptional regulation may drive Hsp70 induction in these muscles through enhanced translation of pre-existing mRNA.

Previous studies examining the Hsp70 response in skeletal and cardiac muscle have reported a more pronounced elevation in Hsp70 gene and protein expression in males compared with females, particularly within 24 h of stress exposure (Shinohara et al., 2004; Paroo et al., 2002a; Paroo et al., 2002b). This pattern is consistent with the induction of Hsp70 protein expression in the male soleus after 24 h and gene expression after 48 h in the female soleus in the present study. Differences in basal *Hsp70* gene expression resulting in differences in the available pool of mRNA transcripts available for translation may explain the sexually dimorphic response in the soleus. However, this appears unlikely in our study, as basal *Hsp70* gene expression was similar between male and female soleus (see Supplementary Figure S3).

Another explanation is that the antioxidant properties of estrogen (Wang et al., 2018; Baltgalvis et al., 2010; Pellegrino et al., 2022) reduce protein damage to a degree that pre-existing Hsp70 levels are sufficient, thus reducing the need for further HSF-1 activation. While this mechanism could explain our findings, the protein oxidation data from our study indicates that female soleus experienced increased oxidative stress 24 h post-HS, suggesting that other mechanisms likely influence the sexually differential induction of the heat shock response in the soleus. Following exhaustive exercise, reduced HSF-1 binding has been reported in female compared with male myocardium. Estrogen was implicated as a modulator of this, as ovariectomy resulted in comparable HSF-1–HSE binding between male and female rats, while subsequent administration of 17β-estradiol reduced binding back to that observed in intact females (Paroo et al., 2002b). Consistent with this, reduced nuclear levels of phosphorylated (active) HSF-1 have also been reported in female *versus* male myocardium 24 h post-HS (Shinohara et al., 2004). Hsp70 accumulation in the nucleus has been shown to attenuate the heat shock response by reducing HSF-1 activity (Masser et al., 2019; Krakowiak et al., 2018; Kmiecik et al., 2020) and recently, in MCF7 cells, Silveira and colleagues reported that estrogen increases Hsp70 recruitment to the nucleus which subsequently reduces HSF-1 recruitment, implying reduced HSF-1 transcriptional activity (Silveira et al., 2023).

Basal *Hsp70* gene expression in the WG was higher in females than in males (see Supplementary Figure S3), which may explain the lack of transcriptional upregulation in the female WG in response to HS, as sufficient pre-existing mRNA could support the observed increases in protein expression. Unlike the soleus, however, the induction of Hsp70 protein expression in WG did not differ between sexes, suggesting that muscle specific, estrogen-mediated effects may play a role. Indeed, the female rat gastrocnemius has been reported to express lower levels of estrogen receptor mRNA, and likely protein, compared with the soleus (Lemoine et al., 2002), potentially limiting estrogen’s protective effect, leading to comparable oxidative damage to intracellular proteins within the muscle in males and females, and a similar need to activate the heat shock response. Nonetheless, this interpretation remains uncertain, as we did not observe significant changes in protein oxidation in the WG of either sex. Together, these findings highlight that the regulation of Hsp70 in WG may be less influenced by sex hormones and more dependent on basal mRNA transcript availability, contrasting with the soleus and underscoring the muscle-specific nature of stress-inducible mechanisms. Having established that the heat shock response of Hsp70 varies by muscle type and, in some cases, by sex, we next considered whether SLN and PLN might exhibit similar stress-inducible regulation in skeletal muscle.

In the soleus, SLN protein appears to increase in males following HS despite no detectable changes in *Sln* mRNA, suggesting post-transcriptional regulation. By interacting with SERCA, SLN modulates cytosolic Ca^2+^ levels and influences downstream Ca^2+^-dependent signaling pathways such as calcineurin and Ca^2+^/calmodulin-dependent protein kinase II, which in turn regulates mitochondrial biogenesis, myogenesis, fibre type transitions, and potentially SLN protein expression itself (Fajardo et al., 2017; Fajardo et al., 2018; Chambers et al., 2022; Maurya et al., 2018). Perhaps, such downstream Ca^2+^-dependent signaling pathways may perform post-transcriptional regulation of *Sln* mRNA to elevate SLN protein expression in the male soleus.

In contrast, SLN protein was undetectable in the WG, despite a late (48 h) rise in *Sln* gene expression in males. Given that basal *Sln* gene expression in WG is much lower compared to soleus (see Supplementary Figure S3), SLN protein levels may not have reached detectable thresholds within 48 h, or expression may require longer or more severe stress. The lack of an SLN response in the female soleus and WG suggests a potential sex difference in the response of this protein to cellular stress. One possible explanation is the influence of estrogen. In the heart, estrogen acting through estrogen receptor-beta, has been shown to modulate Hsp70 mRNA and protein levels (Yu et al., 2006; Voss et al., 2003), thereby altering the heat shock response (Shinohara et al., 2004). It is possible that estrogen could similarly modulate SLN gene and protein expression, attenuating its response to HS in the female soleus and WG. While direct links between estrogen and SLN regulation have not yet been established, our findings highlight this as an important avenue for future investigation.

We have previously shown that, like SLN, PLN can also preserve SERCA structure and function during heat stress (Fu et al., 2020). PLN and SLN are homologous proteins that are capable of regulating both SERCA isoforms, and given the observed induction of SLN protein expression in the male soleus, we hypothesized that PLN would also be upregulated following HS. However, PLN gene expression remained unchanged, and we were unable to detect PLN protein in either muscle. Given our lab’s prior success detecting PLN protein in mouse skeletal muscle using the same methods, we anticipated similar results in rat muscle. The inability to detect PLN protein here is consistent with challenges reported by other groups (Babu et al., 2007), suggesting either that PLN protein expression is extremely low or absent in rat soleus and WG, similar to the low or absent SLN protein expression we observed in the WG. Taken together, Hsp70 and SLN mRNA and protein responded in a sex- and muscle-specific manner to mild *in vivo* HS, while PLN remained largely unchanged. These results highlight the nuanced, context-dependent regulation of stress-responsive proteins.

There are a few limitations to this study–i) The *in-vivo* HS protocol utilized here only elicited mild stress, and perhaps with increased severity, we may have seen even greater changes. ii) We did not assess oxidation of SERCA directly to assess if our HS protocol directly affected SERCA. Perhaps the mild stressor was capable of generating localized oxidative stress around the SR and considering that SERCA is prone to oxidation, it may have been oxidized directly in our stress model. iii) We were unable to assess SERCA function in these samples because our use of glass homogenizer did not allow for us to optimally homogenize muscle for an assay that is very sensitive to sample prep. Future work should explore the roles of sex hormones, more severe stress paradigms, and direct assessment of SERCA integrity and function to clarify the protective contributions of SLN and PLN in skeletal muscle.

## Data Availability

The datasets presented in this study can be found in online repositories. The names of the repository/repositories and accession number(s) can be found below: https://doi.org/10.6084/m9.figshare.30361717.
