# Supplementary material for: Sarcolipin but not phospholamban responds to in-vivo heat stress in rat skeletal muscle similar to the 70-kDa heat shock protein
Source: Front Physiol. 2026 Mar 3;17:1763668. doi: 10.3389/fphys.2026.1763668 (PMC12996975; doi:10.3389/fphys.2026.1763668)
Supplement: Supplementary file 1 [file Supplementaryfile1.docx]

Supplementary Material

# Supplementary Figures and Tables

## Supplementary Figure 1


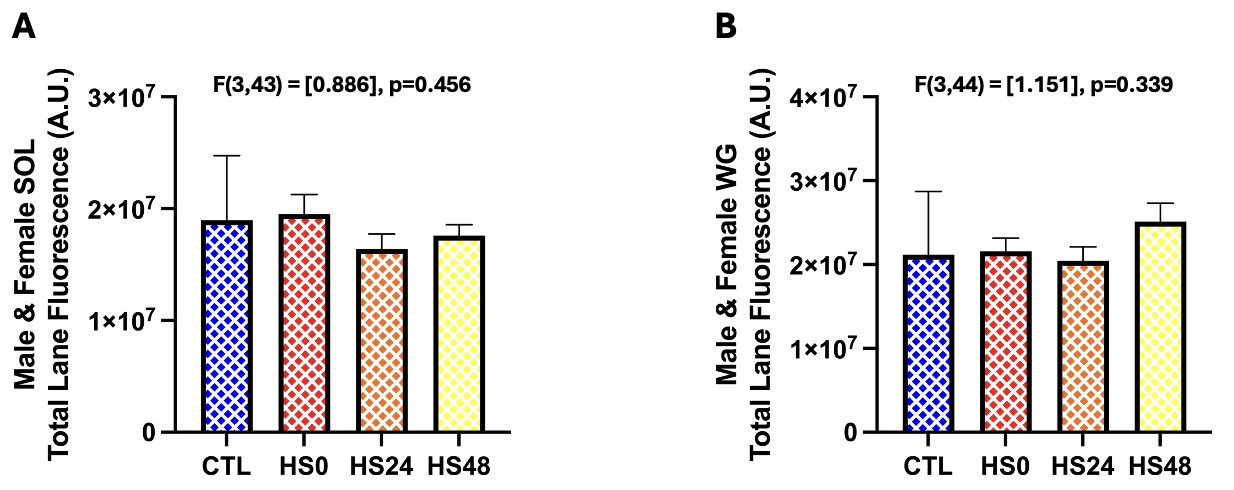


**Supplemental Figure 1: Sex-combined analysis of total protein oxidation under basal conditions and following HS as assessed by IRDye 800CW bound maleimide in rat soleus (SOL) and white gastrocnemius (WG).** **(A-B)** Total protein oxidation in control and HS groups across 3 timepoints in male and female SOL **(A)** and WG **(B)**; n=11-12 per group. Protein oxidation status is reported as total lane fluorescence of the IRDye 800CW bound maleimide to free (reduced) sulfhydryl groups. One-way ANOVA was performed and all data are presented as mean$\pm$SEM.

## Supplementary Figure 2


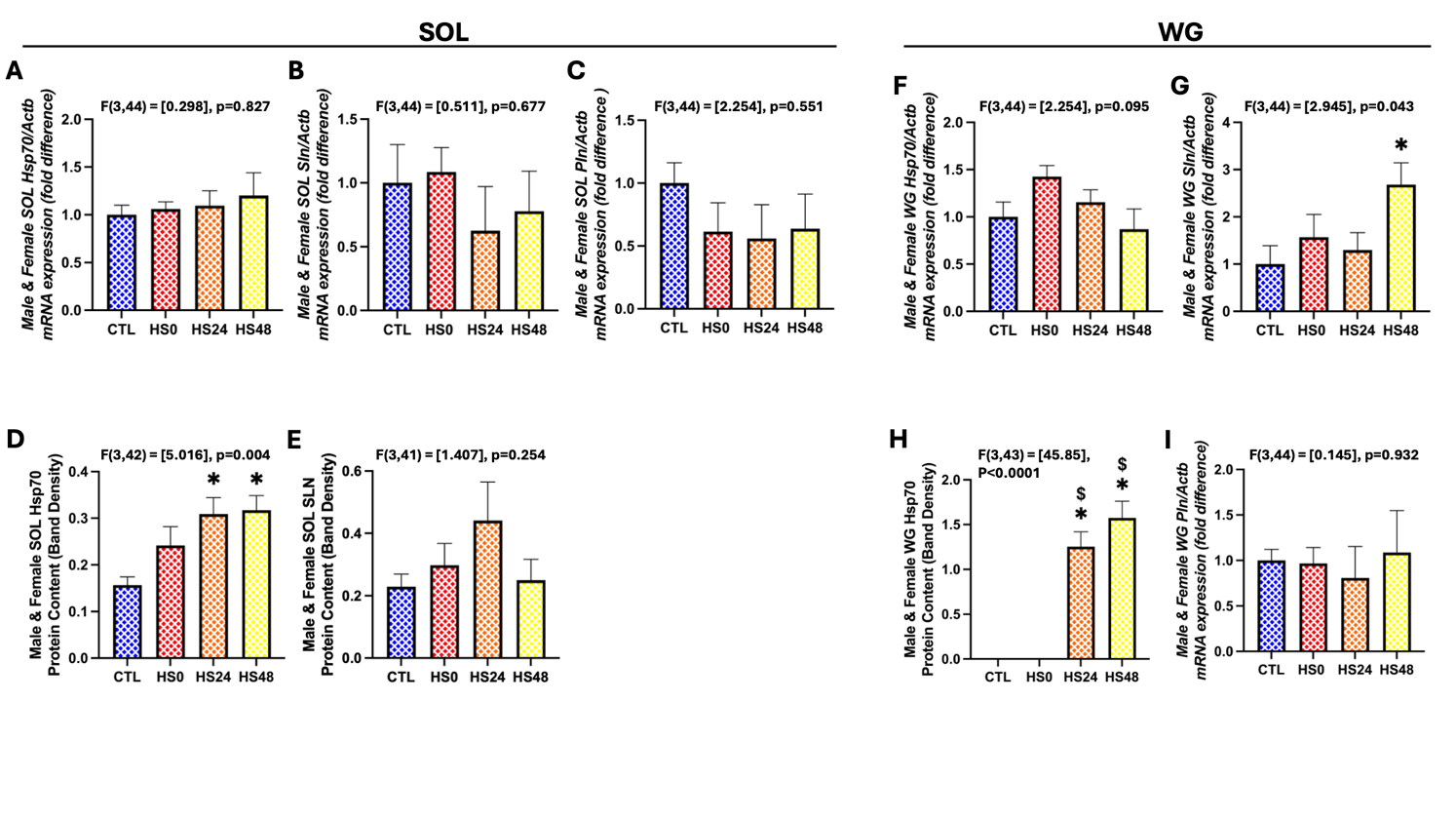


**Supplemental Figure 2: Sex-combined analysis of Hsp70, SLN, and PLN gene and protein response to HS in rat soleus (SOL) and white gastrocnemius (WG).** **(A-E)** Male and female SOL Hsp70 **(A)**, SLN **(B)**, and PLN **(C)** gene (n=12 per group) and Hsp70 **(D)** and SLN **(E)** protein response (n=11-12 per group). **(F-I)** Male and female WG Hsp70 **(F)**, SLN **(G)**, and PLN **(I)** gene (n=12 per group) and Hsp70 protein **(H)** response (n=11-12 per group). One-way ANOVA was performed and all data are presented as mean$\pm$SEM. Gene expression data is represented as fold difference relative to control (CTL). ($) p<0.05 vs HS0; (*) p<0.05 vs CTL.

## Supplementary Figure 3

**
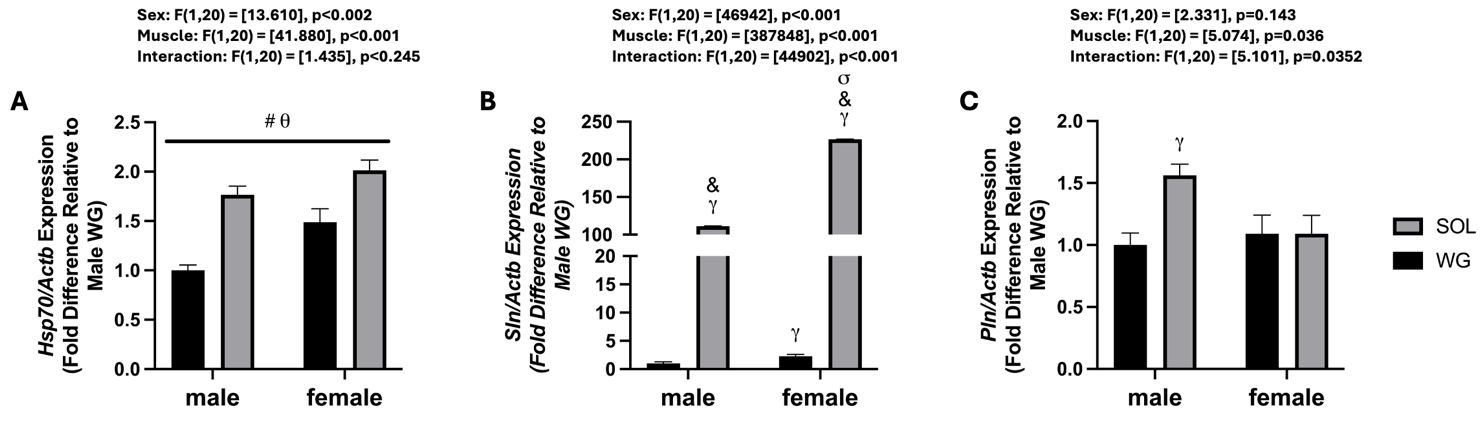
**

**Supplemental Figure 3: Basal gene expression of *Sln, Pln, and Hsp70* in male and female rat soleus (SOL) and white gastrocnemius (WG).** Gene expression data are represented as fold difference relative to WG. Two-way ANOVA was performed and all data are presented as mean$\pm$ SEM. (#) main effect of sex. ($\theta$) main effect of muscle. (&) p<0.05 vs female WG. ($\gamma$) p<0.05 vs male WG. ($\sigma$) p<0.05 vs male SOL.

## Supplementary Table

**Supplementary Table 1.** TaqMan® gene expression assay IDs

| Gene | Organism | Assay ID |
| --- | --- | --- |
| *Hsp70* | Rat | Rn00596544_m1 |
| *Pln* | Rat | Rn01434045_m1 |
| *Sln* | Rat | Rn02769377_s1 |
| *ß-Actin* | Rat | Rn00667869_m1 |
